# Comparison of All-Cause Mortality Between Individuals With COVID-19 and Propensity Score–Matched Individuals Without COVID-19 in South Korea

**DOI:** 10.1093/ofid/ofab057

**Published:** 2021-02-01

**Authors:** Tak Kyu Oh, In-Ae Song, Kyoung-Ho Song, Young-Tae Jeon

**Affiliations:** 1 Department of Anesthesiology and Pain Medicine, Seoul National University Bundang Hospital, Seongnam, South Korea; 2 Division of Infectious Diseases, Department of Internal Medicine, Seoul National University Bundang Hospital, Seoul National University College of Medicine, Seongnam, South Korea; 3 Department of Anesthesiology and Pain Medicine, College of Medicine, Seoul National University, Seoul, South Korea

**Keywords:** infections, population, public health, viruses

## Abstract

**Background:**

We compared all-cause mortality between individuals in South Korea with and without coronavirus disease 2019 (COVID-19) using propensity score (PS) matching.

**Methods:**

This population-based cohort study used data from the National Health Insurance Service COVID-19 cohort database. In the database, we included individuals (COVID-19 patients, control population, and test-negative individuals) aged 20 years or older, regardless of hospitalization. The primary end point was all-cause mortality between January 1, 2020, and August 27, 2020.

**Results:**

A total of 328 374 adults were included in the study: 7713 and 320 660 in the COVID-19 group and the control group. After PS matching, a total of 15 426 individuals (7713 per group) were included in the analysis. All-cause mortality was 3.2% (248/7713) and 1.6% (126/7713) in the COVID-19 group and the control group, respectively. In Cox regression analysis after PS matching, the risk of death in the COVID-19 group was twice as high (hazard ratio, 2.00; 95% CI, 1.61–2.48; *P* < .001) as that in the control group. Among patients aged ≥60 years, the COVID-19 group had a 2.32-fold higher all-cause mortality compared with the control group, while statistically significant differences were not observed in the age groups 20–39 years (*P* = .339) and 40–59 years (*P* = .562).

**Conclusions:**

In South Korea, all-cause mortality was twice as high among individuals with COVID-19 as among those with similar underlying risks, primarily because of the elevated COVID-19-associated mortality in those aged ≥60 years. Our results highlight the need for prevention of COVID-19 with respect to mortality as a public health outcome.

On March 11, 2020, the World Health Organization declared coronavirus disease 2019 (COVID-19) a pandemic [1]. As of October 16, 2020, 38 825 968 cases of COVID-19 and 1 096 833 COVID-19-related deaths have been reported globally [2], and there is no effective and safe vaccine for COVID-19 [3]. Therefore, COVID-19 is still a global public health crisis.

The COVID-19 death rate is reported to vary by country [4], and the case fatality ratio was estimated to be 0.02 (or 2%) by a previously conducted meta-analysis [5]. Moreover, the infection fatality rate has been reported to be 0.95% in the United States [6], and a recent study reported that about 6% of the global population had died due to COVID-19 [7]. However, most studies have focused on mortality rates among COVID-19 patients [5, 7, 8], and previous studies have not compared all-cause mortality among persons diagnosed with COVID-19 with propensity score (PS)–matched controls who were not diagnosed with COVID-19. In addition to individuals diagnosed with COVID-19, the pandemic may affect health-related outcomes even among individuals without COVID-19 [9–11]. Elective procedures have been canceled or delayed, and access to outpatient clinics has been restricted to preserve hospital beds and intensive care unit capacity during the COVID-19 pandemic. Moreover, an unexpected decline in the number of patients seeking emergency medical care was reported during the early phase of the COVID-19 pandemic [12, 13]. Another study also reported that the COVID-19 pandemic has had a negative, widespread, and persistent impact on ST elevation myocardial infarction care in United States [14]. Thus, the risk of all-cause mortality among individuals diagnosed with COVID-19 needs to be compared with that of individuals who were not diagnosed with COVID-19.

Therefore, this study aimed to compare all-cause mortality between individuals in South Korea with COVID-19 and individuals without COVID-19 using PS matching. We hypothesized that individuals with COVID-19 might have higher all-cause mortality than individuals without COVID-19, because COVID-19 might increase the risk of death among individuals with COVID-19.

## METHODS

### Study Design and Ethical Statement

This population-based observational study was conducted and reported according to the Reporting of Observational Studies in Epidemiology guidelines [15].

### Patient Consent Statement

The study protocol was approved by the Institutional Review Board of Seoul National University Bundang Hospital (X-2004-604-905) and the Health Insurance Review and Assessment Service (NHIS-2020-1-291). Informed consent was waived because the data analyses were performed retrospectively using deidentified data derived from the South Korean NHIS database.

### NHIS-COVID-19 Cohort Database and Study Population

The NHIS-COVID-19 cohort database was developed for medical research purposes in cooperation between the NHIS and the Korea Centers for Disease Control and Prevention (KCDC). The KCDC provided data on patients diagnosed with COVID-19 between January 1, 2020, and June 4, 2020, such as COVID-19 diagnosis confirmation date, treatment results, and demographic information. The COVID-19 patients in the NHIS COVID-19 database included all patients who were confirmed as positive by COVID-19 test regardless of hospitalization; therefore, COVID-19 patients who were admitted to the hospital with severe symptoms as well as COVID-19 patients with no or mild symptoms were included in the database. In South Korea, patients who were diagnosed with COVID-19 were admitted to the hospital if they had severe symptoms or conditions such as pneumonia. However, if they had mild or no symptoms, they were isolated and closely monitored in government-managed centers. The COVID-19 patients who are currently undergoing hospital treatment were not included in this database because their treatment outcomes have not yet been determined. Using the data on COVID-19 patients, the NHIS extracted the control population using stratification methods regarding age, sex, and place of residence as of February 2020. The NHIS-COVID-19 cohort database contains disease diagnoses according to the International Classification of Diseases (ICD)–10 codes and prescription information concerning drugs and/or procedures from 2015 to 2020. Finally, the NHIS-COVID-19 database also provides data regarding individuals who were tested for COVID-19 but were found to be negative. Therefore, the NHIS-COVID-19 database comprised 3 groups: COVID-19 patients, control population, and test-negative individuals. We included all individuals (COVID-19 patients, control population, and test-negative individuals) aged 20 years or older and excluded those with incomplete medical records. The control population and test-negative individuals were defined as the control group in this study, because a larger population is needed to identify our main outcome robustly, and the PS matching option served as a method of adjustment to ensure that the characteristics of the 2 groups (COVID-19 patients and the control group) were similar. For this study, an independent medical record technician at the NHIS center, unaffiliated with the study, extracted the data on June 26, 2020.

### Exposure Variable: Confirmation of COVID-19 Diagnosis

The exposure variable in this study was confirmation of COVID-19 diagnosis between January 1, 2020, and June 4, 2020. In South Korea, patients who were diagnosed with COVID-19 were admitted to the hospital if they had severe symptoms such as pneumonia. However, if they had mild or no symptoms, they were isolated and closely monitored in certain government-managed centers.

### End Points

The primary end point of this study was all-cause mortality among all populations in the NHIS-COVID-19 database. It was evaluated from January 1, 2020, to August 27, 2020. All-cause mortality was defined as death due to any reason.

### Covariates

The variables extracted as potential confounders included demographic characteristics (age and sex), annual income level during 2020, place of residence (Seoul, Gyeonggi-do, Daegu, Gyeongsangbuk-do, and other areas), the degree of underlying disability in 2020 (mild and moderate to severe), and Charlson Comorbidity Index (CCI), which was calculated based on the registered ICD-10 diagnostic codes (Supplementary Table 1) from January 1, 2015, to December 31, 2019. Study subjects were categorized into 7 groups according to age: 20–29, 30–39, 40–49, 50–59, 60–69, 70–79, and ≥80 years.

### Statistical Analyses

The baseline characteristics of the participants are reported as frequencies with percentages for categorical variables and means and their SDs for continuous variables. First, we performed propensity score (PS) matching, used to reduce confounders in observational studies, using the nearest neighbor method with a 1:1 ratio, without replacement, and a caliper width of 0.25 [16]. Logistic regression analysis was performed to calculate PS in a logistic model, and all covariates were included in the propensity score model. The absolute standardized mean difference (ASD) was used to determine the balance between the COVID-19 group and the control group before and after PS matching. ASDs between the 2 groups were set below 0.1 to determine whether the 2 groups were well-balanced through PS matching. After confirming adequate balance between the 2 groups, we performed Cox proportional hazards regression analysis for all-cause mortality in the PS-matched cohort. In this Cox regression analysis, the event was defined as any mortality between January 1, 2020, and August 27, 2020, and survival time was calculated as the time from January 1, 2020, until the date of death or until August 27, 2020, for survivors. Considering that Cox regression analysis was a time-to-event analysis, we also performed logistic regression analysis for all-cause mortality in the PS-matched cohort as the primary sensitivity analysis. By performing logistic regression analysis as the primary sensitivity analysis, we determined the odds of all-cause mortality among the COVID-19 group compared with the control group without considering survival time.

As a secondary sensitivity analysis, we fit a multivariable Cox regression model for all-cause mortality for the entire NHIS-COVID-19 cohort in order to (1) determine whether the results obtained from the PS-matched cohort were generalizable to the entire cohort and (2) determine the risk of all-cause mortality among the COVID-19 group with other important covariates in context, not in isolation. All covariates were included in the multivariate Cox model for adjustment, and the CCI and comorbidities that were used to calculate it were included in a different model to avoid multicollinearity. Finally, we performed subgroup analyses according to age and CCI because age and comorbidities were expected to affect mortality in the COVID-19 group [17]. It was confirmed that there was no multicollinearity in all multivariable models involving the entire cohort, with a variance inflation factor of <2.0. The results of the Cox regression are presented as hazard ratios (HRs) with 95% confidence intervals, and those of the logistic regression analysis are presented as odds ratios (ORs) with 95% CIs. C-statistics were used to identify the C-index of the multivariable Cox regression model. All statistical analyses were performed using R software (version 3.6.3 with R packages; the R Project for Statistical Computing, Vienna, Austria). A *P* value <.05 was considered statistically significant.

## RESULTS

### Study Population

**Figure 1.**
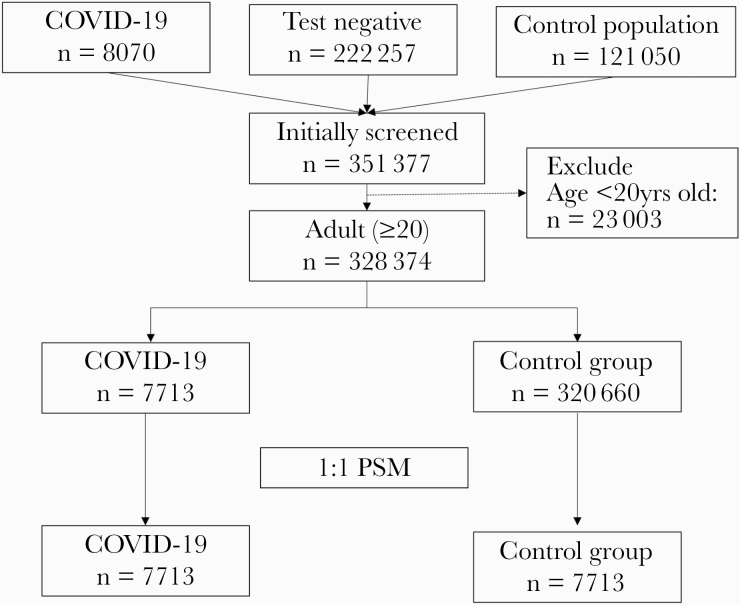
Flowchart depicting selection process for study subjects. Abbreviations: COVID-19, coronavirus disease 2019; PSM, propensity score matching.

The NHIS-COVID-19 cohort comprised 8070 individuals diagnosed with COVID-19, 222 257 test-negative individuals, and 121 050 individuals in the control population; thus, 351 377 individuals were initially screened. Then, 23 003 individuals were excluded as they were <20 years old. Of the remaining 328 374 adult individuals, 7713 belonged to the COVID-19 group and 320 660 to the control group. After PS matching, a total of 15 426 individuals (7713 individuals in each group) were included in the analysis (Figure 1). The results of the comparison of baseline characteristics between the COVID-19 group and the control group before and after PS matching are presented in Table 1. All ASDs between the 2 groups were <0.1 after PS matching, reflecting adequate balance between the 2 groups through PS matching. The distribution of PS also became similar through PS matching (Supplementary Figure 1).

**Table 1.** Comparison of Baseline Characteristics Between COVID-19 Group and Control Group Before and After Propensity Score Matching

	Before Propensity Score Matching			After Propensity Score Matching		
Variable	COVID-19 (n = 7713)	Control (n = 320 660)	ASD	COVID-19 (n = 7713)	Control (n = 7713)	ASD
Sex, male	3048 (39.5)	142 710 (44.5)	0.102	3048 (39.5)	3084 (40.0)	0.010
Age, y						
20–29	2057 (26.7)	68 361 (21.3)		2057 (26.7)	2052 (2.7)	
30–39	832 (10.8)	50 457 (15.7)	0.160	832 (10.8)	835 (10.8)	0.002
40–49	1036 (13.4)	46 901 (14.6)	0.035	1036 (13.4)	1048 (13.6)	0.005
50–59	1567 (20.3)	52 598 (16.4)	0.097	1567 (20.3)	1552 (20.1)	0.012
60–69	1199 (15.5)	43 884 (13.7)	0.051	1199 (15.5)	1200 (15.6)	0.007
70–79	617 (8.0)	31 342 (9.8)	0.065	617 (8.0)	625 (8.1)	0.002
≥80	405 (5.3)	27 117 (8.5)	0.143	405 (5.3)	401 (5.2)	0.001
Annual income level in 2020						
Q1 (lowest)	2439 (31.6)	74 362 (23.2)		2439 (31.6)	2333 (30.2)	
Q2	1445 (18.7)	62 782 (19.6)	0.181	1445 (18.7)	1495 (19.4)	0.017
Q3	1577 (20.4)	77 265 (24.1)	0.021	1577 (20.4)	1643 (21.3)	0.021
Q4 (highest)	2135 (27.7)	100 814 (31.4)	0.091	2135 (27.7)	2112 (27.4)	0.007
Unknown	117 (1.5)	5437 (1.7)	0.084	117 (1.5)	130 (1.7)	0.014
Residence at 2010						
Seoul	510 (6.6)	53 559 (16.7)		510 (6.6)	541 (7.0)	
Gyeonggi-do	431 (5.6)	57 348 (17.9)	0.535	431 (5.6)	479 (6.2)	0.027
Daegu	5036 (65.3)	96 291 (30.0)	0.741	5036 (65.3)	4964 (64.4)	0.020
Gyeongsangbuk-do	933 (12.1)	26 338 (8.2)	0.119	933 (12.1)	960 (12.4)	0.011
Other area	803 (10.4)	87 124 (27.2)	0.549	803 (10.4)	769 (10.0)	0.014
Underlying disability						
Mild degree	318 (4.1)	16 790 (5.2)	0.056	318 (4.1)	288 (3.7)	0.020
Moderate to severe	293 (3.8)	12 073 (3.8)	0.002	293 (3.8)	277 (3.6)	0.011
Charlson comorbidity index	2.7 (2.7)	3.5 (3.4)	0.275	2.7 (2.7)	2.8 (2.8)	0.016
Hypertension	1889 (24.5)	99 768 (31.1)	0.154	1889 (24.5)	1943 (25.2)	0.016
Myocardial infarction	222 (2.9)	12 162 (3.8)	0.055	222 (2.9)	225 (2.9)	0.002
Congestive heart failure	503 (6.5)	36 058 (11.2)	0.191	503 (6.5)	497 (6.4)	0.003
Peripheral vascular disease	1314 (17.0)	64 626 (20.2)	0.083	1314 (17.0)	1353 (17.5)	0.013
Cerebrovascular disease	899 (11.7)	49 746 (15.5)	0.120	899 (11.7)	890 (11.5)	0.004
Peptic ulcer disease	3180 (41.2)	150 619 (47.0)	0.117	3180 (41.2)	3229 (41.9)	0.013
DM without chronic complication	1893 (24.5)	94 401 (29.4)	0.114	1893 (24.5)	1929 (25.0)	0.011
DM with chronic complication	591 (7.7)	32 084 (10.0)	0.088	591 (7.7)	599 (7.8)	0.004
Renal disease	183 (2.4)	16 134 (5.0)	0.175	183 (2.4)	185 (2.4)	0.002
Hemiplegia or paraplegia	140 (1.8)	7095 (2.2)	0.030	140 (1.8)	150 (1.9)	0.010
Rheumatic disease	745 (9.7)	35 463 (11.1)	0.047	745 (9.7)	731 (9.5)	0.006
Mild liver disease	3376 (43.8)	150 617 (47.0)	0.065	3376 (43.8)	3451 (44.7)	0.020
Moderate to severe liver disease	33 (0.4)	3057 (1.0)	0.081	33 (0.4)	36 (0.5)	0.006
Chronic pulmonary disease	3961 (51.4)	186 747 (58.2)	0.138	3961 (51.4)	4072 (52.8)	0.029
Any cancer	602 (7.8)	48 899 (15.2)	0.278	602 (7.8)	633 (8.2)	0.015
Metastatic solid tumor	70 (0.9)	9198 (2.9)	0.207	70 (0.9)	62 (0.8)	0.011
HIV/AIDS	9 (0.1)	647 (0.2)	0.025	9 (0.1)	8 (0.1)	0.004

Presented as mean value with SD or number with percentage.

Abbreviations: ASD, absolute standardized mean difference; COVID-19, coronavirus disease 2019; DM, dibetes mellitus.

### Survival Analysis

Table 2 shows the results of the survival analysis before and after PS matching. After PS matching, all-cause mortality in the COVID-19 group was 3.2% (248 of 7713), while that of the control group was 1.6% (126 of 7713). Cox regression analysis found that the risk of all-cause mortality was twice as high in the COVID-19 group compared with the control group (HR, 2.00; 95% CI, 1.61–2.48; *P* < .001). Logistic regression analysis showed a similar tendency (OR, 1.97; 95% CI, 1.59–2.44; *P* < .001). In the multivariable Cox regression model for all-cause mortality in the entire NHIS-COVID-19 cohort, the COVID-19 group showed a 2.11-fold higher all-cause mortality compared with the control group (HR, 2.11; 95% CI, 1.85–2.40; *P* < .001) (Table 3). The C-index of the multivariable model was 0.90 (95% CI, 0.89–0.90), showing high predictability for all-cause mortality in the multivariable model.

**Table 2.** Mortality From January 1, 2020, to August 27, 2020

		Cox or Logistic Regression	
Variable	No. of Events (%)	Hazard Ratio (95% CI)	*P* Value
Before PSM			
Control	11 318 of 320 660 (3.5)	1	
COVID-19	248 of 7713 (3.2)	0.90 (0.79–1.02)	.097
After PSM			
Control	126 of 7713 (1.6)	1	
COVID-19	248 of 7713 (3.2)	2.00 (1.61–2.48)	<.001
Sensitivity analysis (LR)		OR (95% CI)	
Control	126 of 7713 (1.6)	1	
COVID-19	248 of 7713 (3.2)	1.97 (1.59–2.44)	<.001

Abbreviations: COVID-19, coronavirus disease 2019; LR, logistic regression; OR, odds ratio; PSM, propensity score matching.

**Table 3.** Multivariable Cox Regression Model for Mortality in 2020 Among the Entire NHIS-COVID-19 Cohort

	Multivariable Model	
Variable	Hazard Ratio (95% CI)	*P* Value
COVID-19 (vs control)	2.11 (1.85–2.40)	<.001
Age, 10-y increase	2.01 (1.98–2.05)	<.001
Sex, male (vs female)	1.64 (1.58–1.70)	<.001
Annual income level in 2020		
Q1 (lowest)	1	
Q2	0.96 (0.91–1.02)	.170
Q3	0.90 (0.85–0.95)	<.001
Q4 (highest)	0.80 (0.77–0.84)	<.001
Unknown	1.14 (0.98–1.32)	.091
Residence at 2010		
Seoul	1	
Gyeonggi-do	1.08 (1.03–1.14)	.004
Daegu	0.34 (0.32–0.37)	<.001
Gyeongsangbookdo	0.63 (0.58–0.68)	<.001
Other area	1.04 (0.99–1.09)	.132
Underlying disability		
Mild degree (vs no disability)	1.00 (0.95–1.06)	.927
Moderate to severe (vs no disability)	1.33 (1.25–1.41)	<.001
Charlson comorbidity index, 1-point increase (in other model)	1.10 (1.10–1.11)	<.001
Hypertension	1.12 (1.06–1.18)	<.001
Myocardial infarction	0.98 (0.92–1.054)	.530
Congestive heart failure	1.19 (1.14–1.25)	<.001
Peripheral vascular disease	0.90 (0.86–0.93)	<.001
Cerebrovascular disease	0.94 (0.90–0.98)	.008
Peptic ulcer disease	0.89 (0.85–0.92)	<.001
DM without chronic complication	1.11 (1.06–1.16)	<.001
DM with chronic complication	1.05 (1.01–1.10)	.033
Renal disease	1.12 (1.07–1.19)	<.001
Hemiplegia or paraplegia	1.23 (1.15–1.32)	<.001
Rheumatic disease	0.88 (0.84–0.93)	<.001
Mild liver disease	0.97 (0.93–1.02)	.238
Moderate to severe liver disease	1.76 (1.60–1.94)	<.001
Chronic pulmonary disease	0.88 (0.84–0.92)	<.001
Any cancer	1.56 (1.49–1.63)	<.001
Metastatic solid tumor	2.85 (2.70–3.01)	<.001
HIV/AIDS	0.96 (0.68–1.35)	.821

C-index: 0.90 (0.89–0.90).

Abbreviations: COVID-19, coronavirus disease 2019; DM, diabetes mellitus; NHIS, National Health Insurance Service.

### Subgroup Analyses

Table 4 shows the results of the subgroup analyses according to age and CCI. The COVID-19 group showed a 2.32-fold higher all-cause mortality compared with the control group among individuals aged ≥60 years (HR, 2.32; 95% CI, 2.03–2.65; *P* < .001), while the difference in the risk of all-cause mortality was not statistically significant among individuals in the age groups of 20–39 years (*P* = .339) and 40–59 years (*P* = .562) compared with the control group.

**Table 4.** Subgroup Analyses According to Age Group and Charson Comorbidity Index

	Multivariable Model	
Variable	Hazard Ratio (95% CI)	*P* Value
Age: 20–39 y (n = 121 707, mortality = 219)		
COVID-19 (vs control)	0.38 (0.05–2.74)	.339
Age: 40–59 y (n = 102 102, mortality = 1362)		
COVID-19 (vs control)	1.14 (0.72–1.81)	.562
Age ≥60 y (n = 104 564, mortality = 9985)		
COVID-19 (vs control)	2.32 (2.03–2.65)	<.001
Charson comorbidity index: 0–2 (n = 171 035, mortality = 1297)		
COVID-19 (vs control)	1.75 (1.25–2.45)	.001
Charson comorbidity index ≥3 (n = 157 338, mortality = 10 269)		
COVID-19 (vs control)	2.16 (1.88–2.48)	<.001

Abbreviation: COVID-19, coronavirus disease 2019.

## DISCUSSION

This population-based cohort study showed that there was a 2-fold increase in the risk of all-cause mortality among COVID-19 patients, compared with a PS-matched COVID-19-negative control group. Notably, this higher all-cause mortality is almost entirely attributable to the elevated all-cause mortality in those aged ≥60 years. To our knowledge, this is the first study to report an increase in all-cause mortality associated with COVID-19 in direct comparison with non-COVID-19 causes of all-cause mortality.

In South Korea, the annual mortality was reported as 0.57% in 2019 by Statistics Korea (http://kostat.go.kr/portal/eng/index.action). However, the all-cause mortality rate among the control group after PS matching was 1.6% (126/7713) through August 27, 2020, in this study. This suggests that COVID-19 patients were a sick group to begin with, so the individuals in the PS-matched control group were also in poorer health than people in the general population of South Korea.

Many previous studies have reported the factors associated with increased mortality among COVID-19 patients, and the underlying comorbid status was associated with a higher risk of all-cause mortality among COVID-19 patients [18, 19]. In this study, we adjusted for many confounders, including CCI and comorbidities, using PS matching or multivariable Cox regression modeling to estimate the independent effect of COVID-19 on all-cause mortality in the South Korean population. According to the NHIS-COVID-19 database, the all-cause mortality rate among those confirmed as having COVID-19 through June 4, 2020, was 3.2% (28 of 7713) (Table 2). This was lower than the potential COVID-19-related global mortality of 6% [7]. Despite the lower all-cause mortality among South Korean COVID-19 patients, COVID-19 increases the risk of all-cause mortality; thus, our results justify the effective prevention of COVID-19 infection in the future by vaccination or by wearing a mask.

The results of subgroup analyses according to age are important in this study. A previous study reported that older age is one of the known risk factors for increased mortality among COVID-19 patients, in addition to other risk factors such as male sex, smoking, and underlying comorbid diseases [20]. Thus, the prevention, isolation, and treatment of COVID-19 infection in elderly individuals have become an important public health issue in the COVID-19 pandemic [21]. In this study, we showed that COVID-19 infection increases all-cause mortality among elderly people, with an HR of 2.16 (95% CI, 1.88–2.48).

Although all-cause mortality was not elevated in COVID-19 infection in the younger age group (20–59 years old) compared with the PS-matched younger control group, prevention of COVID-19 infection in this group is still important for 2 reasons. First, as rapid asymptomatic transmission of COVID-19 during the incubation period demonstrated strong infectivity among young COVID-19 patients [22], older people can be affected by transmission from young people. Second, clinical sequelae are commonly reported among COVID-19 survivors, including young people [23]. Third, there might be some cases who died due to COVID-19 without being diagnosed with COVID-19 among the PS-matched control group. Although we did not report the hospitalization rate for all individuals in this study, there were a few cases of prehospital death due to COVID-19 before June 4, 2020, in South Korea. Thus, the impact of hospitalization in COVID-19 patients and PS-matched controls might be limited in this study. Therefore, lack of elevated mortality among those aged <60 years is not the only consideration for future prevention and management.

The impact of CCI on the association between all-cause mortality and COVID-19 infection was also notable in this study, because underlying comorbidities are well-known risk factors for increased mortality among COVID-19 patients [18, 19]. Our study showed that COVID-19 infection increased the risk of all-cause mortality regardless of comorbid status (HR, 1.75 in the CCI 0–2 group; HR, 2.16 in the CCI ≥3 group). This suggests that COVID-19 is associated with a higher risk of all-cause mortality in both healthy and unhealthy adult populations. Therefore, our results suggest that the prevention of COVID-19 infection should be emphasized regardless of comorbid status.

This study has some limitations. First, some important variables, including body mass index and lifestyle factors such as history of smoking and alcohol consumption, were not included in the analysis because they were not available in the NHIS-COVID-19 database. Second, both PS matching and multivariable adjustment are known to reduce the number of known and measured confounders. Therefore, there may be some residual confounding that might have affected the study results. Third, to calculate the CCI, we defined comorbidities using ICD-10 codes. However, the diseases specified by the ICD-10 codes may differ from the actual underlying diseases in our study population. Fourth, since the NHIS-COVID-19 database did not provide the cause of death, the proportion of COVID-19-related deaths among COVID-19 patients was not evaluated in this study. Lastly, the all-cause mortality was evaluated from January 1, 2020, to August 27, 2020; therefore, the results may have been different if we had been able to assess mortality over a longer period.

In conclusion, using the NHIS-COVID-19 database, we showed that all-cause mortality among patients with COVID-19 was twice that of those with similar underlying risks, regardless of hospitalization. This higher all-cause mortality is almost entirely attributable to the elevated mortality in those aged ≥60 years. Our results highlight the need for prevention of COVID-19 with respect to mortality as a public health outcome.

## Supplementary Data

Supplementary materials are available at *Open Forum Infectious Diseases online*. Consisting of data provided by the authors to benefit the reader, the posted materials are not copyedited and are the sole responsibility of the authors, so questions or comments should be addressed to the corresponding author.

ofab057_suppl_Supplementary_Figure_S1

ofab057_suppl_Supplementary_Table_S1
