# Supplementary figures and images for: Comparison of All-Cause Mortality Between Individuals With COVID-19 and Propensity Score–Matched Individuals Without COVID-19 in South Korea
Source: Open Forum Infect Dis. 2021 Feb 1;8(4):ofab057. doi: 10.1093/ofid/ofab057 (PMC7928610; doi:10.1093/ofid/ofab057)

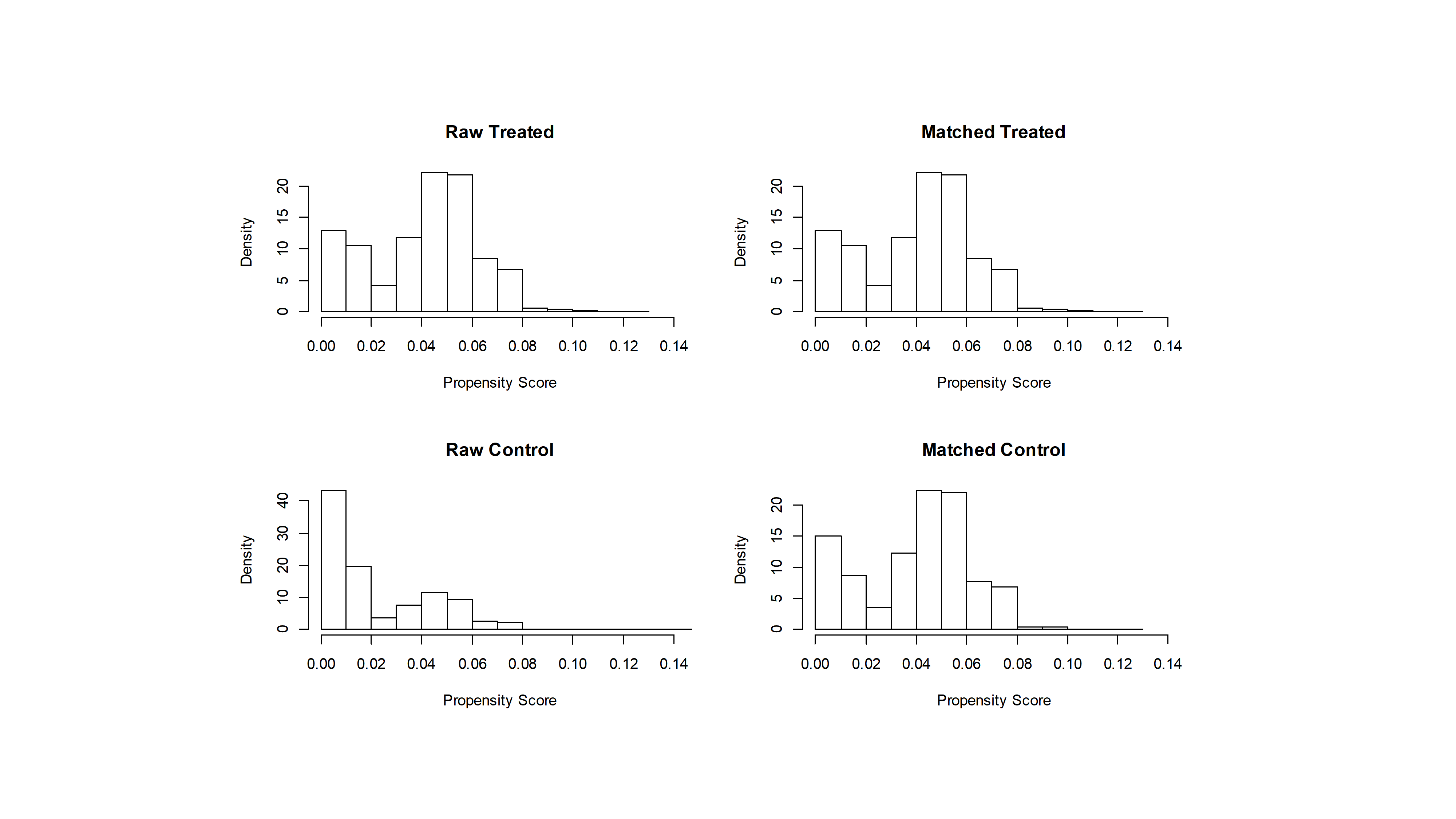

Supplement: ofab057_suppl_Supplementary_Figure_S1 [file ofab057_suppl_supplementary_figure_s1.png]
